# DriverFuse: An R package for analysis of next-generation sequencing datasets to identify cancer driver fusion genes

**DOI:** 10.1371/journal.pone.0262686

**Published:** 2022-02-03

**Authors:** Shikha Roy, Dinesh Gupta

**Affiliations:** Translational Bioinformatics Group, International Centre for Genetic Engineering and Biotechnology, New Delhi, India; King Faisal Specialist Hospital and Research Center, SAUDI ARABIA

## Abstract

We developed the *DriverFuse* package to integrate orthogonal data types such as Structural Variants (SV) and Copy Number Variations (CNV) to characterize fusion genes in cancer datasets. A fusion gene is reported as a driver or passenger fusion gene, based on mapping SV and CNV profiles. *DriverFuse* generates a fusion plot of fusion genes with their mapping SV, CNV profile, domain architecture and classification of its role in cancer. The analysis facilitates discrimination of driver fusions from passenger fusions. To demonstrate the utility of *DriverFuse*, we analyzed two datasets, one each for CCLE (Cancer Cell Line Encyclopedia) for lung cancer and HCC1395BL for breast cancer. The analysis validates the driver fusion genes that are already reported for the datasets. Thus, *DriverFuse* is a valuable tool for studying the driver fusion genes in cancers, enabling the identification of recurrent complex rearrangements that provide intuitive insights into disease driver events.

## Introduction

The availability of cheaper sequencing datasets for cancer projects has led to new opportunities to study the disruptions in cancer genomes. A wealth of information has already been gathered from such projects. However, the molecular mechanism of various SVs in cancer genomes and underlying biology is still unclear [1]. Copy Number Variation (CNV) is also a crucial genomic aberration associated with the molecular etiology of cancer. For example, the deletion of mono-ADP-ribosylhydrolase 2 alters DNA repair and DNA damage checkpoints in human Colorectal Cancer (CRC) [2]. One of the salient abnormalities in the cancer genome is chromosomal rearrangements, resulting in the joining of 2 unrelated genes in the chromosome to produce a fusion gene. The most well-characterized and illustrative example of a fusion gene is the Philadelphia chromosome 12 that joins the N-terminus of BCR (Breakpoint cluster region) with the tyrosine kinase domain of ABL13 (Abelson). The resulting chimeric protein activates tyrosine kinase activity and transforms benign tissue into malignant [3]. The surge in availability of genome sequencing datasets has led to increased identification of genomic rearrangements such as fusion transcripts, copy number variation and structural variants (SV) [4]. Fusion gene identification tools such as JAFFA, SOAPfuse, etc., predict many fusion transcripts, making deciphering driver fusion genes from the passenger genes arduous. Structural variants can produce chimeric mRNAs encoding novel oncogenic proteins termed fusion mRNAs, which may have altered transcriptional output, transcriptionally induced or repressed due to swapping of 5′ ends [5, 6]. Most prostate cancers result from *TMPRSS2*-*ERG* fusion transcripts formed due to somatic deletions of chromosome 21, suggesting the potential role of CNVs in gene-fusion transcripts formation [7]. CICERO tool ranks candidate driver fusion gene-based exon-to-exon chimeric transcript and extensive annotation [8]. Another tool confFuse, scores driver fusion gene based on manually created structural motifs. Whereas, *DriverFuse* considers the molecular distortion in cancer genomes, such as structural variation and copy number variations, to predict driver fusion genes. Compared to other tools, *DriverFuse* is user-friendly and renders graphical output for straightforward interpretation and molecular implications of the driver fusion genes. *DriverFuse* is a user-friendly R package that classifies a list of input fusion genes into driver or passenger genes, based on mapping SVs and information about changes in copy number variation profile.

The *DriverFuse* tool hypothesizes that the oncogenic driver fusion genes can be differentiated from others by mapping structural variants with copy number profile changes. The hypothesis is supported by the fact that fusion gene formation results in CNVs and SV profile changes and vice-versa in cancer genes. For *DriverFuse* validation, we analyzed fusion genes based on integrated CNVs from WGS, with SVs from RNA-Seq, using CCLE lung cancer datasets and a breast cancer cohort dataset. The fusion genes identified using various algorithms demonstrate that the orthogonal data types allow the identification of novel oncogenic fusion transcripts and reveal valuable insights into their role in cancer progression.

## Materials and methods

**Fig 1:**
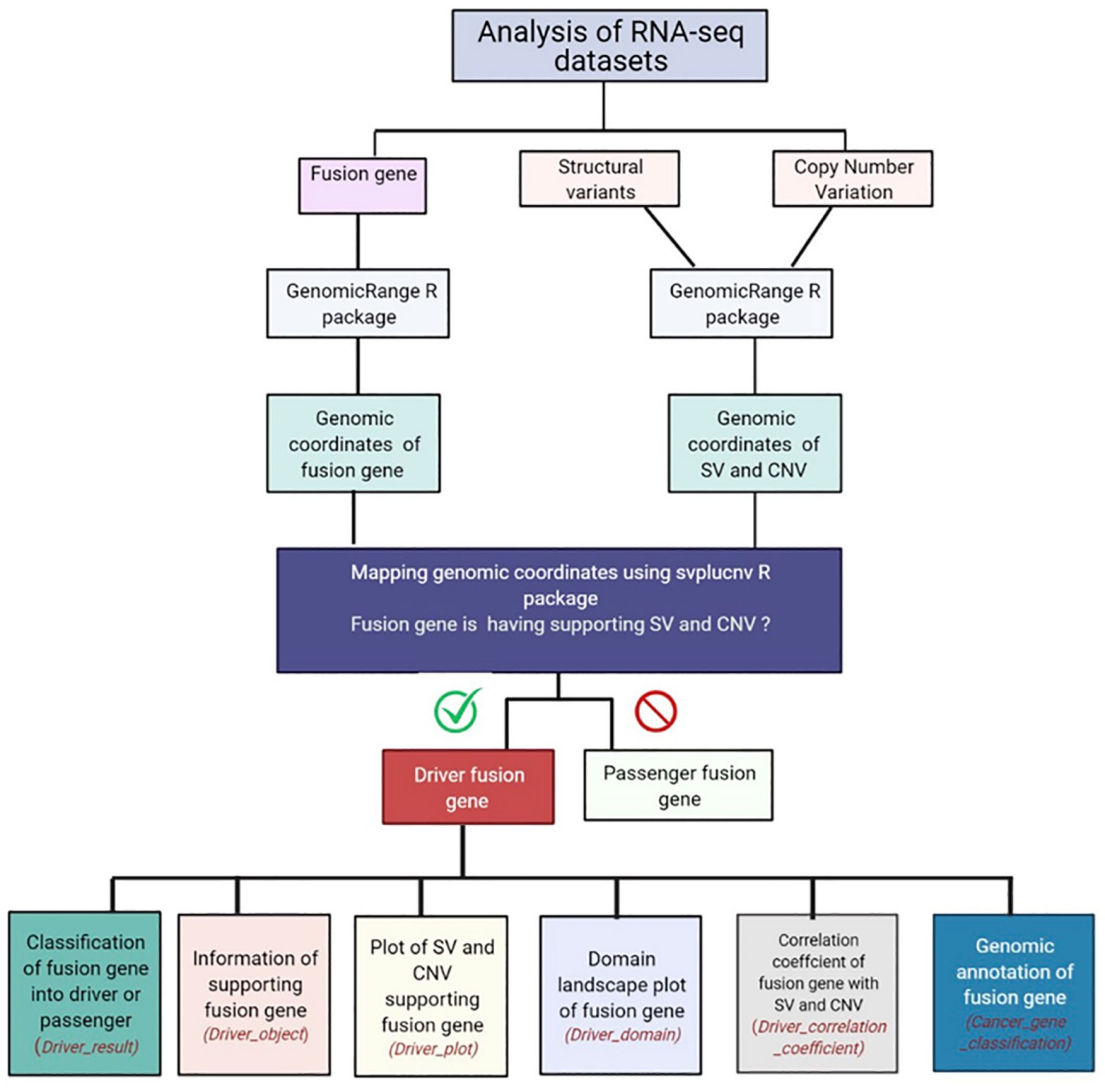
*DriverFuse* workflow. The DriverFuse pipeline consists of several packages. The names of the in-house developed functions are mentioned in italics.

The *DriverFuse* workflow is shown in Fig 1. Summarily, *DriverFuse* takes SV and CNV input files along with a list of fusion genes for a particular sample. It then converts the input CNV and SV into a genomic range object using the genomicRange R package. It then maps the genomic coordinates of the input fusion gene list into SV and CNV, one by one. As our hypothesis is based on the fact that fusion gene formation results in CNV and SV profile changes or vice-versa in cancer genes, the mapping of coordinates of the input fusion gene with SV and CNV is performed (using svpluscnv R package). If a fusion gene has a mapping SV and CNV profile, it is reported as a driver fusion gene, otherwise, it is reported as a passenger gene. Based on this mapping, the tool creates driver object reporting mapping SV classes and CNV profile of the input fusion gene and creates a plot. For a given input fusion gene, *DriverFuse* also calculates the correlation coefficient of the input fusion gene based on mapping SV and CNV profiles. The tool also searches COSMIC (Catalogue of Somatic Mutations in Cancer) cancer census data to classify if the input fusion gene has a role in cancer and details whether it is an oncogene or a TSG (Tumor Suppressor Gene) [9]. It also explores and plots the domain-level landscape of fusion genes using the drawProteins R package.

The *DriverFuse* R package requires three inputs, namely CNV segmentation profiles, SV profiles and a list of input fusion genes. CNV segmentation file includes logR (e.g., log_2_ ratio of the signal of samples). CNVs are derived from RNA-Seq to provide genome-wide gain/loss dosage information. CNV segmentation data, in 6 columns, are required in the following order: sample ID, chromosome (chrom), start location of genomic region profiled (start), end location of genomic region profiled(end), probes ID of sequencing and segmean. CNVs profile segmean value is expressed as log-ratios: e.g.: $2(tumor/normal). In *DriverFuse*, a CNV is expressed as log-ratios: e.g.: $2(tumor/normal). These values may contain copy number states of admixture or subclonal population. SVs are derived from the discordantly aligned reads in RNA-Seq data. SV calls are composed of 8 columns in the following order: sample, chrom1, pos1, strand1, chrom2, pos2, strand2 and svclass. Chrom1 stands for first chromosome and pos1 their genomic position associated with the left position of associated SVs. Chrom2 stands for the first chromosome and pos2 their genomic position associated with the right position of associated SVs. Another value, svclass stands for the input variant classes may include duplication, deletion, inversion, insertion and translocation. This may be obtained from WGS by identifying reads and read-pairs that align discordantly to the reference genome. A list of input fusion genes may be obtained from any fusion gene detection tool. For reference, sample files are included in the package.

### Overview of functionalities in *DriverFuse*

The *DriverFuse* consists of several functions developed for specific tasks. The *DriverFuse* function *Input_svc* creates an input SV file for a user input data to determine if a mapping fusion gene is “Driver” or a “Passenger”. *Input_cn*v function creates an input CNV file for a user input data to determine if a mapping fusion gene is “Driver” or a “Passenger.” *Driver_result_list* classifies list of input fusion genes into “Driver” or “Passenger” fusion genes, based on mapping coordinates of SVs and the CNVs. The *Driver_object* function creates an object of input fusion gene based on their mapping SV and CNV. The function *Driver_plot* plots the mapping SV and CNV profile for input fusion transcripts. The *Driver_correlation_coefficient* function plots the correlation coefficient between mapping SV and CNV profile for a given input fusion gene. The *Cancer_gene_classification* function provides genomic annotation for the driver fusion gene, categorizing its role in cancer along with other features such as tumor and mutation type. The *Driver_domain* function provides genomic feature annotation for driver fusion genes. Exploring the domain-level landscape of fusion genes is important to identify the role of the genes in cancer progression.

## Results

### Analysis of CCLE lung cancer datasets using DriverFuse

**Fig 2:**
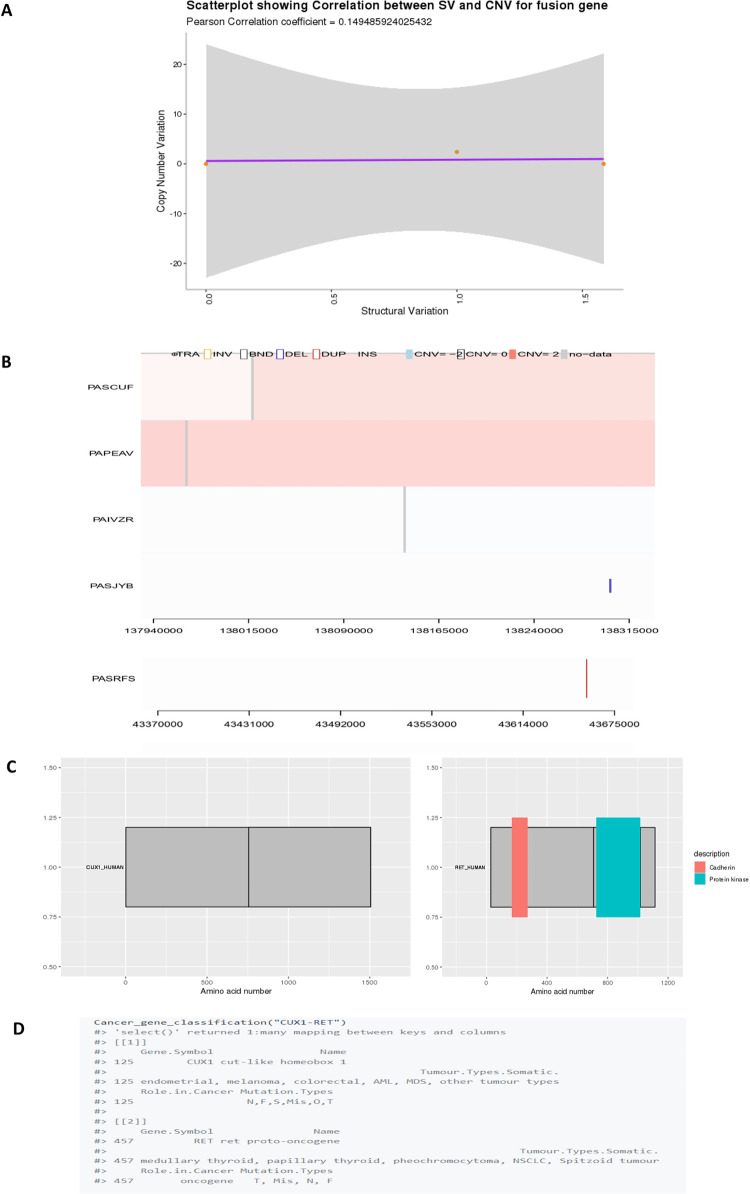
A: *Driver_object* for “CUX1-RET” fusion gene in CCLE lung cancer dataset, B: *Driver_plot* for “CUX1-RET” fusion gene in CCLE lung cancer dataset, C: *Driver_domain* plot for “CUX1-RET” fusion gene in CCLE lung cancer dataset, D: *Driver_correlation_coefficient* for “CUX1-RET” fusion gene in CCLE lung cancer dataset.

To illustrate the utility of *DriverFuse*, we analysed CCLE (Cancer Cell Line Encyclopedia) lung cancer datasets. CCLE is a compilation of gene expression, chromosomal copy number, and massively parallel sequencing data from 947 human cancer cell lines to translate cancer genomic data into knowledge of tumor biology and therapeutic avenues [10]. CCLE also contains information of the established multiple cell lines, human small cell (SCLC) and non-small cell lung cancers (NSCLC), to be utilized by the scientific community worldwide. The SV and CNV dataset for CCLE lung cancer samples were available with the svpluscnv R package [4]. *Driver_result* reports the “CUX1-RET” fusion gene as a driver for the lung cancer sample. *Driver_object* reports SV and CNV profile in the lung cancer sample responsible for the formation of driver fusion gene “CUX1-RET” in NSCLC. *Driver_correlation_coefficient* calculates the correlation coefficient of the input fusion gene “CUX1-RET” in the lung cancer sample (Fig 2A). *Driver_plot* visualizes supporting SV and CNV profile in the lung cancer sample for driver fusion gene “CUX1-RET” (Fig 2B). *Driver_domain* plots domain information of the driver fusion gene “CUX1-RET” (Fig 2C). ‘*Cancer_gene_classification*’ provides annotation of the “CUX1-RET” fusion gene and classifies them as “oncogene” (Fig 2D). All the sample files are also available in the tool vignette file (https://github.com/tbgicgeb/DriverFuse).

### Analysis of HCC Breast cancer cell line datasets using *DriverFuse*

To give another example of *DriverFuse* usage, we analyzed the HCC1395BL breast cancer sequencing datasets. HCC1395BL corresponds to the B lymphoblastoid cell line of a 43-year-old white female with TNM stage 1, grade 3 primary ductal carcinoma and a prior history of cancer. We obtained the breast cancer sample datasets from the NCBI bio project with accession number PRJNA201238. They were analyzed by cnvkit to generate CNV profile and speedseq for SV. *Input_svc* is used to convert the HCC1395BL sample SV file into a format for mapping the input fusion gene. Similarly, *input_cnv* is used to convert the HCC1395BL sample CNV file into a format utilized to map to the input fusion gene. *Driver_result* reports the “MYH9-EIF3H” fusion gene as a driver for the HCC1395BL sample. *Driver_object* reports SV and CNV profile in the HCC1395BL sample responsible for the formation of driver fusion gene “MYH9-EIF3H”. *Driver_correlation_coefficient* calculates the correlation coefficient of the input fusion gene “MYH9-EIF3H” in the HCC1395BL sample. *Driver_plot* visualizes supporting SV and CNV profile in the HCC1395BL sample for driver fusion gene “MYH9-EIF3H”. *Driver_domain* plots domain information of the driver fusion gene “MYH9-EIF3H”. All the results are depicted in the tool vignette file (https://github.com/tbgicgeb/DriverFuse). R package version 4.0.5 is required to run the tool.

## Conclusions

Characterization of gene fusions provides valuable insights into their role in cancer causation and pathophysiology. Unlike previously developed tools, *DriverFuse* classifies input fusion genes as driver or passenger fusion genes based on mapping SV and CNV profiles. The utility of *DriverFuse* has been demonstrated by the analysis of two datasets with known driver fusion genes. It predicts the driver fusion genes and provides the information of molecular events responsible for its formation. Additionally, it is a user-friendly R package that provides additional information such as domain architecture and cancer gene classification, which gives clues for its biological mechanism for promoting or accompanying cancer. *DriverFuse* classifies the “CUX1-RET” fusion gene as a driver for the CCLE lung cancer sample. Non-squamous NSCLC (Non-small-cell lung carcinoma) patients exhibit significant clinical benefits when treated with selpercatinib and pralsetinib, against RET gene fusions, demonstrated by LIBRETTO-001 and ARROW clinical trials [11]. This is an example where knowledge of existence of a particular fusion gene is used for prescribing a personalized treatment. In the case of the breast cancer dataset, *DriverFuse* reports the “MYH9-EIF3H” fusion gene as a driver for the HCC1395BL sample. This EIF3H gene was previously discovered with RNA-seq and validated by PCR in breast cancer SK-BR-3 cell line [12]. Hence, in view of correct validations, *DriverFuse* is a valuable tool to explore fusion genes in cancer datasets where driver fusion genes have not been reported.
